# Supplementary figures and images for: Comprehensive analysis of the lysine succinylome in fish oil-treated prostate cancer cells
Source: Life Sci Alliance. 2023 Sep 8;6(11):e202302131. doi: 10.26508/lsa.202302131 (PMC10487806; doi:10.26508/lsa.202302131)

**Fig 3B**

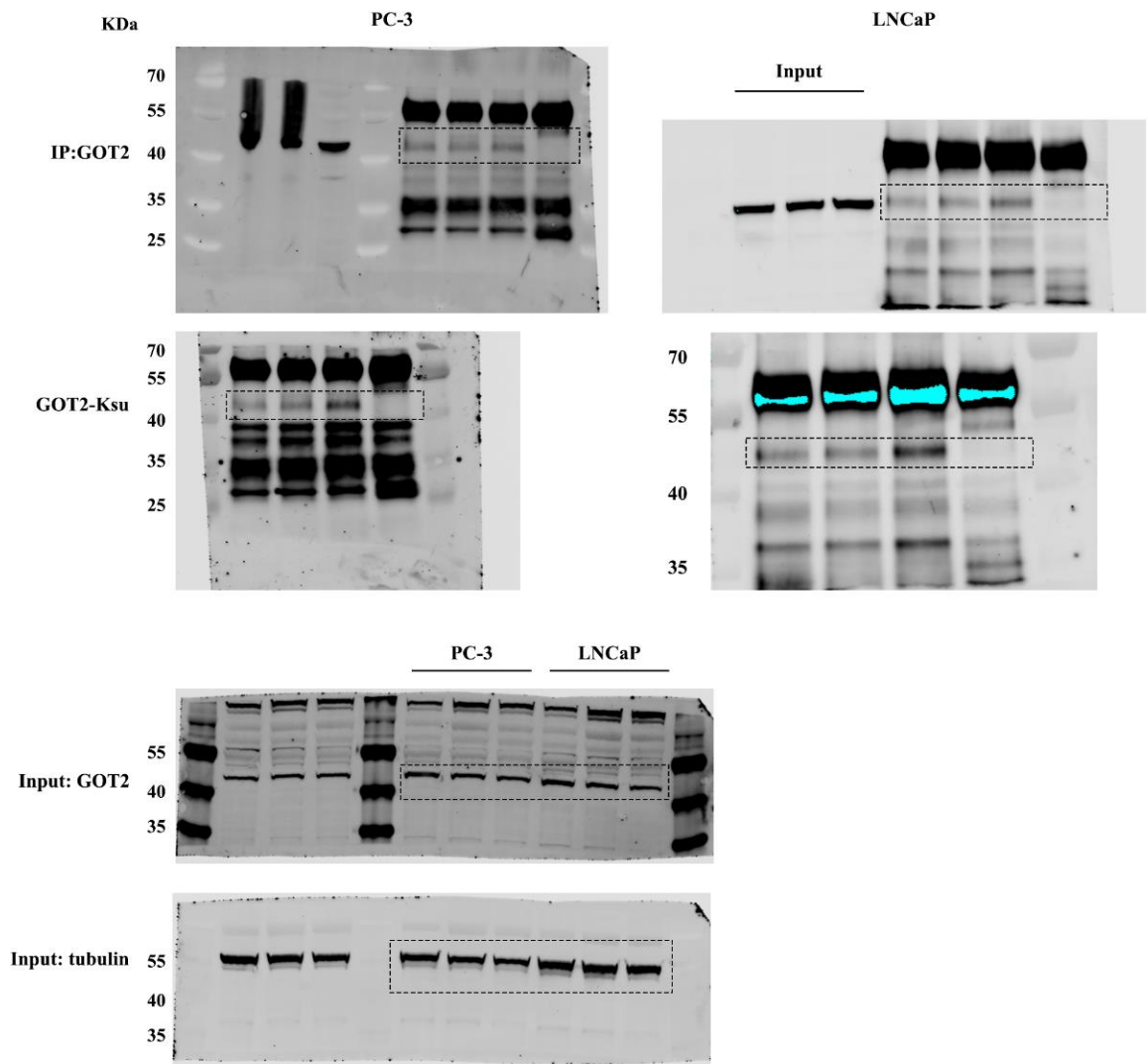

Supplement: Supplementary file 3 [file LSA-2023-02131_SdataF3.pdf]

**Figure 4C**

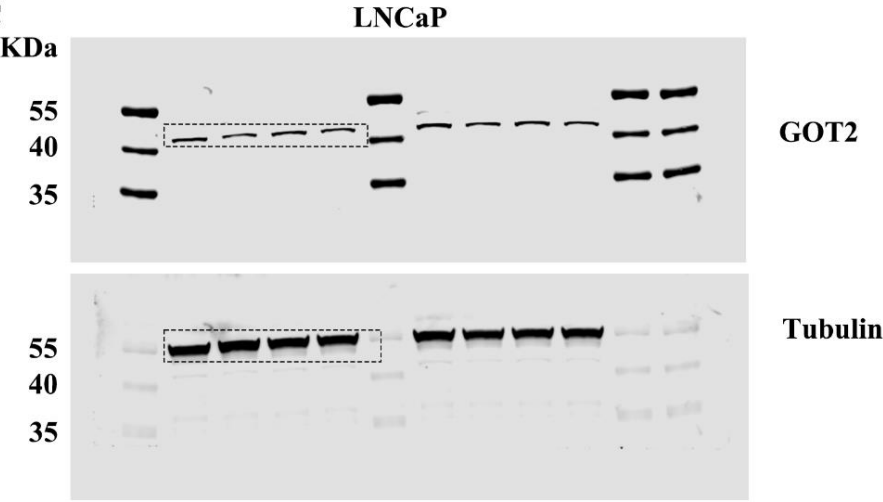

**Figure 4D**

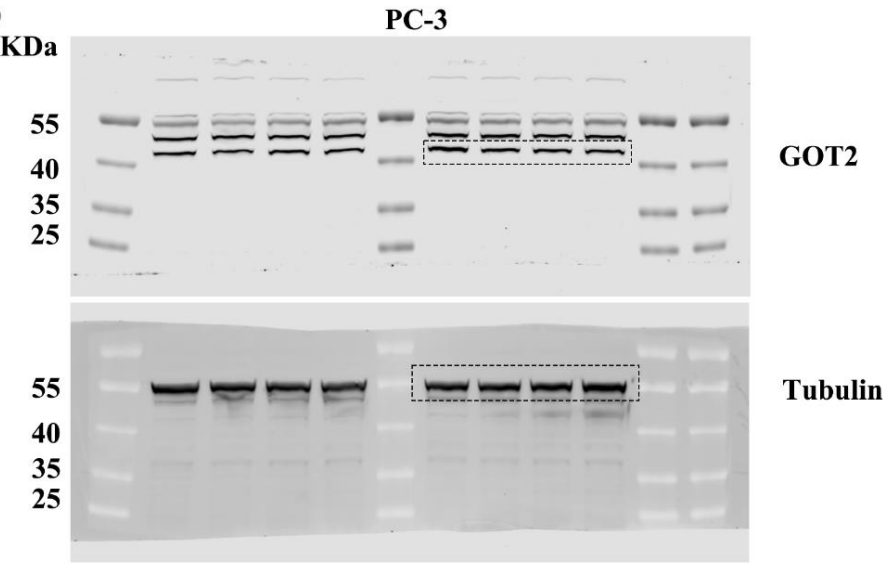

Supplement: Supplementary file 4 [file LSA-2023-02131_SdataF4.pdf]
